# Digital Phenotyping of Voice Biomarkers for Psychological States in College Students: Longitudinal Mobile App Study

**DOI:** 10.2196/103398

**Published:** 2026-11-13

**Authors:** York Li, Tara Maddala, Shreeya Behera, Robert D Henry, Adam Whitney, Jason Carlson, Jeremy P Jamieson

**Affiliations:** 1 School of Information Sciences (iSchool), University of Illinois Urbana-Champaign, Champaign, IL, United States; 2 Pandora Bio, 1669 Hollenbeck Ave Ste 199, Sunnyvale, CA, 94087, United States, 1 408-828-2997; 3 Department of Statistics and Data Science, Northwestern University, Evanston, IL, United States; 4 Tobacco Settlement Endowment Trust (TSET) Health Promotion Research Center, University of Oklahoma, Tulsa, OK, United States; 5 Department of Biostatistics & Epidemiology, University of Oklahoma Health Campus, Tulsa, OK, United States; 6 Department of Psychology, University of Rochester, Rochester, NY, United States

**Keywords:** mental health, students, digital intervention, digital phenotyping, voice, biomarkers

## Abstract

**Background:**

College students face significant mental health challenges, yet traditional assessments often rely on in-person self-reports and long-form self-report survey approaches. The prevalence of smartphones and longitudinal digital phenotyping—using data from devices to rapidly monitor mental health processes—offers an opportunity to assess well-being using a nondisruptive, prevention-focused “light touch” approach via voice journaling and emoji-based expression. While voice biomarkers are associated with clinical anxiety and depression, little research has explored voice biomarkers’ relation to psychological predictors of mental health processes in young adults.

**Objective:**

This study aimed to explore the utility of smartphone-captured voice biomarkers as objective indicators of psychological predictors of affective states in college students.

**Methods:**

College students (N=289) used the Meet Pandora smartphone app for over 2 months. Approximately 3 times per week, participants responded to voice journal prompts, such as “Describe a place you like to visit.” Voice biomarkers were extracted from responses, and their association with concurrent psychological measurements (ie, voice journaling measured closest in time to psychology surveys) was modeled using multivariable, mixed-effects, covariate-adjusted (gender, baseline mental health diagnosis, prompt sentiment, foreign national status, and time) models. Random intercepts were included to control for baseline individual differences, and all analyses were adjusted for multiple comparisons.

**Results:**

Associations were predominantly within students. On the 5-point daily affect scale (higher scores indicate more negative affect), negative affect was characterized by more utterances than usual (odds ratio [OR] 1.16, 95% CI 1.09‐1.23; adjusted *P*<.001) and less positive language (OR 0.92, 95% CI 0.87‐0.97; adjusted *P*=.007), accompanied by reduced vocal energy and pitch: magnitude mean (OR 0.83, 95% CI 0.78‐0.88), pitch mean (OR 0.80, 95% CI 0.73‐0.86), and root-mean-square energy (OR 0.90, 95% CI 0.85‐0.95; all adjusted *P*<.001), with harmonics-to-noise ratio and pitch variance showing the same pattern (both adjusted *P*≤.01). Among students who used more positive language than their peers on average, stress was lower (OR 0.68, 95% CI 0.50‐0.92; adjusted *P*=.049) and their self-regard was higher (adjusted *P*=.04).

**Conclusions:**

While speech and acoustic features did not predict weekly measures of loneliness or acceptance, they were associated with stress, self-regard, and daily affect fluctuations. More positive language was associated with lower stress and higher self-regard. These findings highlight the utility of high-frequency assessments via voice biomarkers as signals of affective changes in college students. This work highlights that brief vocal samples may be able to detect early, affect-specific signs of mental health risks in young adults, which has important implications for psychopathology prevention and improving well-being.

## Introduction

The transition to college represents a critical developmental period characterized by increased independence, shifting social networks, and heightened academic and psychosocial demands. Thus, college transitions are inherently stressful [1-3]. As a result of uncertainty and other stressors, college students carry a substantial mental health burden. Recent data indicate 38% of students screen positive for moderate or severe depression and 34% for anxiety, with 13% reporting suicidal ideation in the past year [4]. In addition to these more severe diagnosable mental health problems, many students experience high levels of negative stress, loneliness, not feeling accepted, and negative affect that may impair functioning and progress to clinical disorders [4,5].

Early detection of negative mental health states represents a critical opportunity for timely, targeted, early intervention that can improve both mental health and academic outcomes [5,6]. Indeed, social isolation and loneliness increase the risk of depression and anxiety up to 9 years downstream [7], and college students who report early and often that they do not belong tend to have higher depression at the end of a semester [8]. Early detection efforts can be particularly impactful in college populations because of stressful transitions these young people undergo during a developmental period when social hierarchies are unstable and affective states are more dynamic and context-dependent relative to older adults [6].

Even though there is a great need for detecting risk for mental health problems in college students, traditional assessments are poorly suited to capturing early signals. Subpar detection can be traced to an overreliance on episodic, in-person evaluations when problems have already escalated to the point of concern and/or retrospective self-report measures administered infrequently and with questionable precision. Additionally, both standard approaches are limited by biases associated with subjective self-reported measurement [6]. Ecological momentary assessment (EMA) research has demonstrated that momentary data collection substantially improves accuracy and ecological validity compared to retrospective reporting, but typical EMA methods disrupt daily routines by “pinging” individuals with prompts to complete self-reports [6]. Thus, “light touch” digital approaches that greatly reduce burdens on college students, but can also collect data in brief time windows, may be more acceptable and engaging to young people, a perspective supported by qualitative evidence [9,10].

Digital mobile devices may provide a low-burden, scalable means for assessing, and ultimately improving, college students’ mental health [11]. In addition to providing access to self-report questionnaires, mobile phones enable digital phenotyping through collection of multimodal data streams, including voice recordings through voice journaling. Voice-derived biomarkers such as prosodic, spectral, and temporal speech features have demonstrated utility in detecting and monitoring depression and anxiety [12,13]. More recent studies using mobile-collected speech data further support the feasibility of predicting mental health symptom severity in real-world populations to detect clinical conditions and estimate severity [13]. These findings suggest that vocal biomarkers may provide an objective and scalable marker of psychopathology. However, existing research has focused primarily on identifying or diagnosing clinical conditions, with limited attention to negative affective states or to young adult populations. This blind spot is particularly notable given that strong and volatile negative emotions predict the onset of mood disorders [8]. From a psychopathology prevention perspective, it is critical to identify the risk markers of a pending depressive or anxious episode.

Although prior work has examined the use of voice biomarkers in relation to individual mental health constructs, no study, to our knowledge, has evaluated their ability to predict multiple psychological state changes (eg, perceived stress, loneliness, perceived acceptance, and core affect) simultaneously within a college population. Also, while much research on core affect focuses on specific emotions as predictors of downstream outcomes [14], advances in emotion science have emphasized understanding more basic underlying affective processes that contribute to the construction of emotional experiences [15]. That is, researchers frequently emphasize core affect—or low-level, consciously accessible affective feelings and neurophysiological responses [16]—states of valence (positivity vs negativity) and activation (high vs low) as proximal predictors of health-related outcomes [17]. The current work advances research at the nexus of mobile sensing, emotion, and affective states by focusing on identifying biomarkers associated with core affect as assessed via emoji usage.

To advance knowledge in identifying affective risk factors, the research reported here leveraged a mobile health platform, Meet Pandora (developed by Taehyung Cho), to collect high-frequency, multimodal data including ecologically relevant self-report and voice-derived features from college students in their daily environments. We evaluated whether these signals predict psychological state changes in stress, loneliness, acceptance, and core affect, with the goal of enabling earlier detection of mental health risk in this high-need population.

## Methods

### Participants

Participants were undergraduate students enrolled at a highly selective private Northeast US university and recruited through a university study portal. All research was approved by the site’s institutional review board, along with informed consent obtained from all participants. Two cohorts of students were recruited: (1) spring 2024 semester (Cohort 1, n=169) and (2) spring 2025 semester (Cohort 2, n=120). Data from both cohorts were integrated for analysis, with most self-report surveys and voice prompts being the same in both studies. Participants in Cohort 2 were offered a stress survey that was not available to the participants in Cohort 1, and Cohort 2 also had additional voice prompts that students were able to respond to (Multimedia Appendix 1).

### The Meet Pandora App

The Meet Pandora smartphone app, available on Android and Apple iOS operating systems, was used to collect data. After signing informed consent, per protocol, students were onboarded onto the app and were asked to review and accept Pandora Bio’s privacy policy. Additionally, before submitting any data on the app, they were prompted to enable the app to use the device microphone and were asked to opt in (check) other permissions for location data collection and use of their smartphone camera. Voice recordings were not collected passively and required the students to actively engage (click a button) to record. They were told that all data would be deidentified and that their identifiable data would not be shared. Meet Pandora facilitates frequent, low-burden self-report using formats such as voice journals, self-reports, and emoji-based representations of core affect. The tool collects data both actively through participant self-reports (validated instruments such as the Patient Health Questionnaire 8-item (PHQ-8), the Generalized Anxiety Disorder 7-item scale (GAD-7), and the Flourishing and Stress scales, as well as brief daily, weekly, and monthly self-reports), and passively through derived voice biomarkers, GPS data, and phone usage metrics (Table 1).

**Table 1.** Data types, collection methods, and assessment frequencies of the meet Pandora smartphone application.

Data type	Collection method	Frequency	Description
PHQ-8^a^ and GAD-7^b^	Active	Monthly	Validated surveys measuring depression and anxiety.
Flourishing, acceptance, loneliness, stress, and self-regard	Active	Weekly	Self-reports assessing stress, flourishing, peer acceptance, loneliness, and overall self-regard.
Emoji-based affect	Active	Daily	Selection of 1‐3 emojis representing current affect, mapped to valence and arousal.
Voice journals	Active input and passive derivation	Daily (prompted 3 × per week)	30‐120–second audio responses to prompts; used to passively extract acoustic and speech biomarkers.
GPS data	Passive	Daily	Continuous background collection of location data.
Phone usage metrics	Active	Daily	Manually uploaded logs of device usage and interaction behaviors.

a PHQ-8: Patient Health Questionnaire 8-item.

b GAD-7: Generalized Anxiety Disorder 7-item scale.

### Self-Reported Measures

#### Acceptance and Loneliness

Questions on acceptance and loneliness were administered to participants weekly. To measure acceptance, participants were asked, “How accepted do you feel by your friend group?” (1=not at all accepted and 5=strongly accepted). To measure loneliness, they were asked, “How often have you felt lonely?” (1=very slightly or not at all and 5=extremely). A high or low acceptance and loneliness dichotomous label was derived from participants’ responses, where a score of 3 (the midpoint) or higher was labeled as “high” acceptance or loneliness, and scores of 1 or 2 were labeled as “low.”

#### Stress

As part of the weekly survey, participants reported their stress by responding to the question “How much stress are you experiencing in your life right now? (1=none, 4=a moderate amount, and 7=an extreme amount).” An ordinal stress label was derived from participants’ responses by categorizing scores into 3 ranked groups: low (scores 1‐3), medium (score 4), and high (scores 5‐7). Notably, this stress measure was only included in the second study (n=120).

#### Self-Regard

Another essential psychological state that was measured and derived is the participant’s self-regard [18]. As part of the weekly self-reports, participants were asked these four questions: “How accepted do you feel by your friend group?” “How often have you felt lonely?” “To what extent did you go out of your way to avoid one or more difficult situations?” and “Overall, how good or bad did you feel about yourself this week? (1=Extremely good to 7=Extremely bad).” A composite weekly self-regard score was derived in 2 ways. First, a weekly continuous self-regard score was derived by averaging z-score-scaled responses to the 4 questions (excluding observations for which at least one question was unanswered). Second, for interpretability, self-regard weekly state was derived as high or low self-regard using principal component analysis (PCA) and K-means clustering.

#### Daily Affective States

Participants also provided their affective states via emojis during daily prompts. Participants chose up to 3 out of 16 emojis to best represent how they felt in the moment. Available options (and their activation and valence categorization) were: Accepted (low activation and positive), Angry (high activation and negative), Annoyed (high activation and negative), Anxious (high activation and negative), Confused (low activation and negative), Contented (low activation and positive), Excited (high activation and positive), Fearful (high activation and negative), Happy (high activation and positive), Hopeful (high activation and positive), Lonely (low activation and negative), Proud (high activation and positive), Refreshed (low activation and positive), Relaxed (low activation and positive), Sad (low activation and negative), and Tired (low activation and negative; Figure 1). Because every participant’s daily entry included at least 1 and up to 3 emojis, the first emoji was treated as the primary indicator of their core affect and used to derive all subsequent daily affect labels. To evaluate broad daily affect states, a daily ordinal label was calculated to capture the net polarity of all emojis selected during a given self-report. This was computed by calculating the ratio of positive emojis to the total number of emojis entered, which was then mapped onto a standardized ordinal scale.

We also calculated valence by arousal interactions. The first emoji selected by participants was divided into 4 categories: high activation and positive, high activation and negative, low activation and positive, and low activation and negative. This classification system was created by intersecting the binary valence dimension (positive vs negative affect) with the binary arousal dimension (high vs low activation).

**Figure 1.**
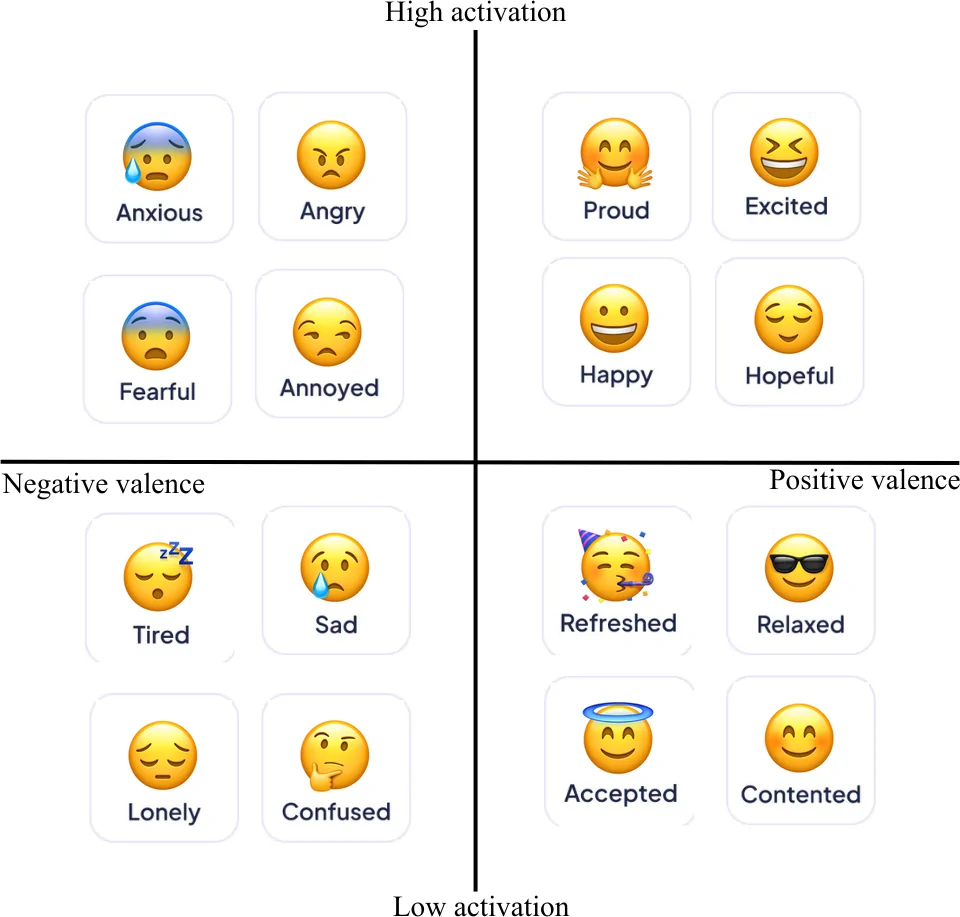
The 16 emojis available for daily-affect self-report, categorized by valence and activation. Students selected up to 3 emojis to describe their momentary affective state. The first emoji students selected was used to derive the daily valence, arousal, and interaction labels in the analysis.

### Passive Measures

Voice biomarkers were derived from participant speech recordings to capture daily variation in vocal and linguistic behavior. We derived features that capture both how they speak and what they say. During the study, participants were prompted to provide a voice journal 3 times per week by responding to 1 of 50 available questions, such as “Describe the highs and lows of your day.” Questions were either positive or neutral, designed to prompt responses between 30 seconds and 2 minutes. Participants could choose a different question to respond to if they did not want to respond to the one initially surfaced to them. From the participants’ audio responses, features from 2 general categories were derived and measured: speech and acoustic. The description of all derived speech and acoustic features is shown in Table 2.

**Table 2.** Definitions of voice features extracted from voice journal recordings.

Feature name	Definition
Speech features	
Word count	Total number of words spoken.
Words per minute	How fast the person is talking.
Number of utterances	How many separate “bursts” of speech occurred (separated by pauses).
Utterances per minute	The rhythm of the conversation, how often they start a new sentence or thought.
Duration (seconds)	How long the entire recording lasts.
VADER^a^ score	Context-aware sentiment measure: “very happy” is stronger than “happy.” [19]
Acoustic features	
Average spectral centroid	The “brightness” of the sound. High values sound “sharp” or “tinny”; low values sound “dark.” [20,21]
Average spectral bandwidth	The “texture” of the sound. Does it use a wide range of frequencies or just a narrow one? [20,21]
Average roll-off frequency	A measure of where energy is distributed, determining if a sound is perceived as deep or bright [20,21].
HNR^b^	The clarity of the voice. A high score means a clear voice; a low score sounds raspy or breathy [22,23].
RMS energy^c^	The physical volume or loudness of the recording [20,21].
Magnitude mean	How loud the signal is on average [20,21].
Magnitude variance	How variable the volume of the signal is [20,21]
Zero crossing rate	How “noisy” the audio is. High rates often indicate hissing, static, or “S” sounds [20,21].
Pitch mean	The average highness/lowness of the voice [22]
Pitch variance	How much the person’s tone “jumps around” [22]

a VADER: Valence Aware Dictionary and Sentiment Reasoner.

b HNR: harmonics-to-noise ratio..

c RMS: root-mean-square.

### Data Analysis Plan

#### Joining Voice Entries and Weekly or Daily Self-Report

Voice data entries were joined to each participant’s corresponding responses to weekly prompts to integrate the biomarker data with psychological labels (loneliness, acceptance, stress, and self-regard). To ensure temporal relevance leading up to the self-reported psychological states, a 7-day lookback window was applied. Daily voice biomarker data were linked to a weekly survey label only if the audio entry was recorded on the same day or within the 7 days immediately preceding that specific self-report. For analysis of voice and daily self-report, the voice and daily affect labels were joined if both data points were recorded on the exact same calendar day for each participant. This day-level alignment ensured that the vocal expressions being analyzed had direct correspondence to the participant’s self-reported affective state at that specific time.

#### Feature Processing, Biomarker Outliers, and Data Cleaning

To understand and remove outliers in the derived voice biomarkers, a large language model (LLM; Google Gemini 2.0 Flash) was used to examine every instance to identify physically impossible observations [24]. Based on the model’s output, 424 outliers were identified due to the presence of background noise. Multimedia Appendix 2 includes chat history and prompts. Table 3 summarizes the removal criteria, which were implemented using R (R Foundation for Statistical Computing) code based on the criteria below.

In the multivariable mixed-effects modeling of voice biomarkers associated with loneliness, acceptance, stress, and daily emojis, a target feature removal was implemented to maximize data retention. For instance, if the mean pitch for a specific voice sample was physically impossible, only the mean pitch for that instance was removed, and the rest of the features for that sample were retained.

**Table 3.** Outlier removal criteria and context, including valid ranges and the number of instances removed for each metric^a^.

Metric	Normal and Valid range	Academic basis
Pitch mean	40‐500 Hz	Ranges outside this for adults usually indicate octave jumps or tracking errors [25]. A total of 333 instances of pitch mean under 40 were removed. No instances were removed on the upper limit.
Speaking rate (WPM^b^)	10‐300 WPM	Values lower than 10 WPM likely indicate prolonged silence (eg, recording left on); human speech articulatory limits; reading rates rarely exceed 275 WPM [26]. A total of 49 instances of WPM under 10 were removed. No instances were removed on the upper limit.
Duration	≥3.0 seconds	Minimum window required for stable acoustic feature estimation [27]. A total of 23 instances of duration under 3 were removed.
Harmonics-to-noise	≥1.0 dB	Lower values indicate a signal dominated by noise rather than vocal periodic energy [23]. A total of 68 instances of HNR^c^ under 1 were removed.
Utterance frequency	≤30 per minute	Rates >30 per minute suggest the voice activity detector is incorrectly chopping noise segments [28]. A total of 10 instances of utterance frequencies above 30 were removed.

a Criteria are not mutually exclusive; a single voice instance could fail more than one threshold (eg, low harmonics-to-noise ratio and an implausible pitch mean from the same noisy recording). The 424 total reflects unique instances removed for at least one criterion, not the sum of the individual counts above.

b WPM: words per minute.

c HNR: harmonics-to-noise ratio.

From participants’ daily voice journals, 16 features were extracted and derived. Prior to statistical modeling, a correlation analysis was conducted to assess voice feature multicollinearity. Magnitude variance, which measures the variance in volume, had a Pearson correlation of 0.92 with magnitude mean, and average spectral centroid, which measures the brightness and sharpness of the audio, had a Pearson correlation of 0.94 with zero crossing rate. Consequently, these 2 features were excluded from subsequent analysis. The feature correlation matrix is given in Multimedia Appendix 3. Prior to modeling, voice biomarkers were standardized using the built-in *scale()* function in R to ensure a mean of 0 and a SD of 1, thereby ensuring the direct comparison of effect sizes across different features.

#### Modeling Process

Because each voice sample was elicited by a prompt that varied in emotional sentiment (positive or neutral), the sentiment of the prompt is a potential source of variation in the voice biomarkers. For example, a positive prompt may elicit a faster speech than a neutral one, introducing variation in the voice biomarker that reflects the eliciting condition rather than the student’s underlying state. Therefore, we evaluated whether prompt sentiment was associated with voice biomarkers, and where it was associated, we adjusted for prompt sentiment to reduce measurement-related variability. Specifically, for each voice biomarker (feature), the model treated prompt sentiment as a binary value (positive or neutral) and was parameterized as follows:


$$logit\left(P\left(PromptSentiment_{ij}=1\right)\right)\;=\;\beta_{0}\;+\;\beta_{1}\;Feature_{ij}\;+\;u_{j}+\varepsilon_{ij}$$

where Prompt_Sentiment_ij_ denotes prompt sentiment for the i^th^ observation from the j^th^ student, Feature_ij_ represents the voice feature for observation i from participant j, u_j_ is the student-level random intercept, and $\varepsilon_{ij}$ is the error term.

Voice biomarker features were also divided into 2 distinct groups: speech and acoustic features. Speech features are features that describe the temporal characteristics. Acoustic features describe the underlying physical properties of the voice, such as pitch mean, root-mean-square (RMS) energy, and magnitude. To control for the inflation of Type I errors from multiple comparisons, *P* values were adjusted using the Benjamini-Hochberg False Discovery Rate method within each feature group (speech and acoustic; Multimedia Appendix 4).

#### Outcome Variable Coding

Weekly psychological states (acceptance and loneliness) were treated as binary (high vs low). Self-regard was modeled 2 ways: as a binary outcome (1=low self-regard and 0=high self-regard) and as a continuous z-score composite, on which higher scores indicate lower self-regard. Daily affect sentiment was treated as a 5-point affect scale, and stress was treated on a 3-point scale with higher numbers associated with higher stress. Valence and arousal derived from emojis as described earlier in the methods were coded as follows: valence was treated as 1=negative and 0=positive, while arousal was treated as 1=high activation and 0=low activation. The combination of valence and arousal was treated as 1=negative and high activation, with 0 used to denote 3 other categories (positive and high activation, positive and low activation, and negative and low activation).

#### Analysis Modeling of Weekly and Daily Outcomes and Voice Biomarkers

Modeling the association between voice biomarkers and daily and weekly outcomes accounted for the repeated outcome measures over days or weeks, repeated voice biomarkers over weeks, the measurement-related variability introduced by the prompt sentiment, and potential confounders (ie, person-level features that are associated with both the outcome of interest and with the voice biomarkers). Each voice biomarker was first standardized and then represented in the model as person-level mean (between-person effect) and person-mean centered by subtracting the person-mean from the value at the given timepoint (within-person effect). Potential confounders that were included in all models include gender identity (male, female, or other), mental health diagnosis (yes or no), and foreign national status (yes or no). In addition to gender identity, mental health diagnosis, and foreign national status fixed effects, additional fixed effects included day number and voice biomarker (feature). Random effects for student (intercept) were also included to account for repeated measures. Consistent with the analysis of prompt sentiment, voice biomarkers were divided into two groups: speech and acoustic, and *P* value adjustments were made within each feature group.

In each model, the psychological state (acceptance, loneliness, stress, self-regard, or daily affect) served as the dependent variable, and a single voice biomarker served as the primary predictor, entered as both its person-level mean (between-student effect) and its person-mean-centered deviation (within-student effect). One model was fit per voice biomarker, each adjusted for the covariates described above and including a random intercept for student.

The model family was determined by the measurement scale of the outcome. Dichotomous outcomes (acceptance, loneliness, and the binary self-regard label) were fit as logistic mixed-effects models using *glmer()* in the *lme4* package. Ordinal outcomes (weekly stress on a 3-point scale and daily affect on a 5-point scale) were fit as cumulative-link, proportional-odds mixed models using *clmm()* in the ordinal package. Continuous weekly self-regard was fit as a linear mixed-effects model using *lmer()* in the *lme4* and *lmerTest* packages, with degrees of freedom and *P* values derived from the Satterthwaite approximation. All models were fit with the *bobyqa* optimizer, and 95% CIs were obtained from the fixed-effects estimates. Associations are reported as odds ratios (OR) for the logistic and ordinal models and as unstandardized regression coefficients for the linear model, in which the outcome is on a standardized (z-score) scale. The logistic parameterization is as follows and the parameterization for the ordinal stress and continuous self-regard outcome model is similar.


$$\begin{array}{ll} \mathrm{logit}\left(P\left(Outcome_{ij}=1\right)\right) & =\beta_{0}+\beta_{1}FeatureMean_{j}+\beta_{2}{FeatureDi\;f\;\;f}_{ij}\\ \;+\beta_{3}Gender_{j}+\beta_{4}Diagnoses_{j}+\beta_{5}ForeignNational_{j}\\ \;+\beta_{6}PromptSentiment_{ij}+\beta_{7}DayNumber_{ij}+u_{0j}+\varepsilon_{ij} \end{array}$$

where Outcome_ij_ denotes the binary outcome variable for the i^th^ observation from the j^th^ student, FeatureMean_j_ represents the mean of the feature for the jth student, and FeatureDiff_ij_ represents the feature difference of the ith observation from the mean for the jth student. Gender_j_ represents the gender, Diagnoses_j_ represents the diagnosis status of the j^th^ student, ForeignNational_j_ represents the foreign national status of the j^th^ student, PromptSentiment_ij_ represents the prompt the student j responded to at observation, i, DayNumber_ij_ represents the response day i of the jth student, and u_0j_ is the student-level random intercept and ε_ij_ is the error term.

### Ethical Considerations

The study was conducted under an institutional review board–approved study protocol at the University of Rochester. The University of Rochester Research Subjects Review Board (RSRB) provided approval for all data collection and data treatment procedures prior to the collection of any data with the RSRB approval number of 00011296. All participants signed informed consent and were enrolled in-person into the study. Participants were informed by the University of Rochester study coordinator that their participation was voluntary and that they could withdraw at any time. All participants were offered the opportunity to ask questions regarding informed consent. Before using the Meet Pandora app, at the time of onboarding, and following informed consent, the Meet Pandora privacy policy and terms and conditions of use were surfaced in the app, and students were asked to read and accept the terms and conditions. The privacy policy states that their data will not be identifiable and will not be shared beyond deidentified data used for research purposes. All participants were required to be 18 years or older at the time of enrollment. Age and gender characteristics are summarized in the paper.

## Results

### Cohort Characteristics

Students (N=289) participated for an average of 49.40 days (SD 20.78, range 1‐101 days). The age and prevalence of mental health diagnoses are representative of a typical college-age population. The cohort is diverse with respect to sexual orientation, race, religion, and first-generation and international students and is skewed towards females (229/289, 79%). Table 4 contains demographic information.

In total, 281 of 289 (97.2%) students responded to 1 or more voice prompts throughout the study. A total of 2036 weekly self-reports were available for analysis. After accounting for missing values, 1330 (65.6%) of entries reported low loneliness, and 696 (34.4%) reported high loneliness. For acceptance, 1893 (93.7%) of entries reported high acceptance, and 128 (6.3%) reported low acceptance. Because the stress measure was only administered to the second cohort, 802 weekly self-reports included stress data. After accounting for missing values, 271 (34.7%) reported low stress, 189 (24.2%) reported medium stress, and 322 (41.2%) reported high stress. For weekly continuous self-regard measurements, 273 students had 1308 entries with a valid derivation. For the dichotomous label of weekly self-regard, 703 (53.8%) indicated high self-regard and 605 (46.2%) indicated low self-regard.

There were significantly more data for daily entries. For daily ordinal affect, there were 5687 entries with 1906 (33.5%) negative, 489 (8.6%) somewhat negative, 530 (9.3%) neutral, 620 (10.9%) somewhat positive, and 2142 (37.7%) positive affect. When categorized by the interaction of valence and arousal, 1810 (31.8%) of these daily entries reflected low-activation and negative states, 1506 (26.5%) low-activation and positive, 1462 (25.7%) high-activation and positive, and 909 (16%) high-activation and negative.

**Table 4.** Cohort characteristics (N=289)^a^.

Characteristic	Cohort 1	Cohort 2	Total
Age (years)			
Mean (SD)	20.13 (1.53)	20.18 (1.45)	20.15 (1.51)
Median (IQR)	20 (19-21)	20 (19-21)	20 (19-21)
Range	18‐28	18‐26	18‐28
Gender, n (%)			
Male	32 (18.9)	16 (13.3)	48 (16.6)
Female	130 (76.9)	99 (82.5)	229 (79.2)
Other	6 (3.6)	4 (3.3)	10 (3.5)
Not disclosed	1 (0.6)	1 (0.8)	2 (0.7)
Sexual orientation, n (%)			
Heterosexual	124 (73.4)	86 (71.7)	210 (72.7)
LGBTQ+^b^	27 (16.0)	28 (23.3)	55 (19)
Other	13 (7.7)	2 (1.7)	15 (5.2)
Not disclosed	5 (3.0)	4 (3.3)	9 (3.1)
Neurodivergent, n (%)			
Yes	9 (5.3)	10 (8.3)	19 (6.6)
No	144 (85.2)	103 (85.8)	247 (85.5)
Not disclosed	16 (9.5)	7 (5.8)	23 (8.0)
First-generation student, n (%)			
Yes	29 (17.2)	21 (17.5)	50 (17.3)
No	138 (81.7)	99 (82.5)	237 (82.0)
Not disclosed	2 (1.2)	0 (0.0)	2 (0.7)
International student, n (%)			
Yes	37 (21.9)	24 (20.0)	61 (21.1)
No	130 (76.9)	96 (80.0)	226 (78.2)
Not disclosed	2 (1.2)	0 (0.0)	2 (0.7)
Religion, n (%)			
Unaffiliated	40 (23.7)	32 (26.7)	72 (24.9)
Christian	35 (20.7)	29 (24.2)	64 (22.1)
Atheist	31 (18.3)	17 (14.2)	48 (16.6)
Other	14 (8.3)	17 (14.2)	31 (10.7)
Hindu	17 (10.1)	6 (5.0)	23 (8.0)
Muslim	11 (6.5)	6 (5.0)	17 (5.9)
Jewish	10 (5.9)	7 (5.8)	17 (5.9)
Not disclosed	11 (6.5)	6 (5.0)	17 (5.9)
Race, n (%)			
Asian	81 (47.9)	49 (40.8)	130 (45)
White	49 (29.0)	40 (33.3)	89 (30.8)
Mixed	14 (8.3)	13 (10.8)	27 (9.3)
Hispanic, Latino/a/x, or Spanish origin	9 (5.3)	8 (6.7)	17 (5.9)
Black	8 (4.7)	7 (5.8)	15 (5.2)
Other	8 (4.7)	3 (2.5)	11 (3.8)
Mental health diagnoses^c^, n (%)			
Non-diagnosis	109 (64.5)	74 (61.7)	183 (63.3)
Anxiety	23 (13.6)	27 (22.5)	50 (17.3)
Depression	18 (10.7)	25 (20.8)	43 (14.9)
Not disclosed	16 (9.5)	7 (5.8)	23 (8.0)
ADHD^d^	7 (4.1)	10 (8.3)	17 (5.9)
Unspecified diagnosis	13 (7.7)	0 (0.0)	13 (4.5)
Other	8 (4.7)	4 (3.3)	12 (4.2)
Time in study (days)			
Mean (SD)	49.22 (21.31)	49.07 (20.2)	49.40 (20.78)
Median (IQR)	53 (38-58)	53 (39-58)	53.0 (38-58)
Range	1‐92	1‐101	1‐101
Self-regard survey contributions (entries)			
Mean (SD)	3.1 (1.5)	6.6 (3.5)	4.8 (3.0)
Median (IQR)	3 (2-4)	7 (4-9)	4 (3-6)
Range	0‐9	0‐16	0 – 16^f^
Loneliness survey contributions (entries)			
Mean (SD)	7.2 (3.5)	6.7 (3.5)	7.3 (3.3)
Median (IQR)	7 (5-9)	7 (4-9)	8 (4-9)
Range	0‐17	0‐16	0 – 17^f^
Acceptance survey contributions (entries)			
Mean (SD)	7.2 (3.6)	6.7 (3.5)	7.2 (3.3)
Median (IQR)	7 (5-9)	7 (4-9)	8 (4-9)
Range	0‐17	0‐16	0 – 17^f^
Stress survey contributions (entries)			
Mean (SD)	—^e^	6.5 (3.6)	6.7 (3.6)
Median (IQR)	—	7 (4-9)	7 (4-9)
Range	—	0‐16	0 – 16^f^
Daily survey contributions (entries)			
Mean (SD)	33.2 (16.8)	32.7 (17.3)	33 (17)
Median (IQR)	33 (22-44)	35.5 (18-44)	34 (20-44)
Range	0‐83	0‐95	0‐95
Prompt sentiment, n (%)			
Positive	2100 (49.8)	1559 (65.6)	3659 (55.5)
Neutral	2113 (50.2)	816 (34.4)	2929 (44.5)
Voice journal contributions (entries)			
Mean (SD)	23.8 (13.2)	19.2 (12.7)	21.9(13.2)
Median (IQR)	22 (14-32)	17 (9-29)	21 (11-31)
Range	1‐60	2‐70	1‐70
Voice journal contributions per week			
Mean (SD)	3.42 (1.22)	2.86 (1.9)	3.19 (1.56)
Median (IQR)	3.37 (2.56-4.25)	2.59 (1.75-3.57)	3.04 (2.19-4.07)
Range	0.5‐7	0.58‐14	0.5‐14

a Percentages are based on the total sample.

b LGBTQ+: lesbian, gay, bisexual, transgender, queer, and others.

c For mental health diagnoses, participants were able to select more than one category; therefore, mental health diagnoses are not mutually exclusive, and cumulative percentages exceed 100%.

d ADHD: attention-deficit-hyperactivity disorder.

e Not applicable.

f When students refreshed the app in some instances, multiple entries per week were captured instead of one entry per week per protocol.

### Prompt Sentiment and Voice Biomarkers

Five speech features returned statistically significant associations (adjusted *P*<.05) with positive prompt sentiment, suggesting that whether the prompt was positive or neutral introduced variation in speech features. Conversely, no acoustic features returned statistically significant results. Because prompt sentiment was shown to affect participants’ speech features, it was added as a controlling covariate in subsequent multivariable analyses. All subsequent models associating voice biomarkers with outcomes converged. Tables 5 and 6 contain speech and acoustic features and their analysis results.

**Table 5.** Analysis results for prompt sentiment and speech features^a^.

Feature	Odds ratio (95% CI)		*P* value	Adjusted *P* value
Word count	1.035 (0.979-1.093)		.23	.23
Words per minute	1.172 (1.108-1.239)		<.001	<.001
Number of utterances	0.851 (0.806-0.899)		<.001	<.001
Utterances per minute	0.933 (0.884-0.985)		.01	.01
Duration (seconds)	0.891 (0.843-0.942)		<.001	<.001
VADER^b^ Score	2.506 (2.336-2.689)		<.001	<.001

a Analysis was based on 281 students with evaluable data. Odds ratios represent the odds of responding to a positive versus a neutral prompt per 1 SD increase in each feature. Higher words per minute and Valence Aware Dictionary and Sentiment Reasoner score are associated with higher odds of having responded to a positive prompt. Higher number of utterances, utterances per minute, and duration are associated with higher odds of having responded to a neutral prompt.

b VADER: Valence Aware Dictionary and Sentiment Reasoner.

**Table 6.** Analysis results for prompt sentiment and acoustic features^a^.

Feature	Odds ratio (95% CI)		*P* value	Adjusted *P* value
Average spectral centroid	0.976 (0.926-1.030)		.38	.76
Average spectral bandwidth	1.038 (0.984-1.096)		.17	.76
Average roll-off frequency	0.991 (0.939-1.045)		.73	.91
RMS^b^ energy	1.049 (0.995-1.106)		.08	.76
Magnitude mean	1.002 (0.950-1.057)		.94	.94
Magnitude variance	1.003 (0.951-1.058)		.90	.94
Zero crossing rate	0.971 (0.921-1.024)		.28	.76
(Harmonics-to-Noise)	0.989 (0.936-1.045)		.70	.91
Pitch mean	0.974 (0.919-1.031)		.36	.76
Pitch variance	0.983 (0.929-1.041)		.57	.91

a Analysis was based on 281 students with evaluable data. Odds ratios represent the odds of responding to a positive versus a neutral prompt per 1 SD increase in each feature. None of the acoustic features were associated with higher odds of being prompted by a positive prompt.

b RMS: root-mean-square.

### Acceptance, Loneliness, Stress, and Self-Regard

Self-regard was modeled in 2 ways, as a continuous outcome and as a dichotomous outcome. Inspection of the distribution of responses across Likert-scale responses for each of the outcomes indicated that dichotomization was necessary for loneliness and acceptance, and that collapsing to 3 categories was required for stress in order to estimate within-student and between-student associations (Multimedia Appendix 5). While a sufficient number of state changes were not observed for modeling the association of voice biomarkers with a change in a student’s state of acceptance (results were not statistically significant), the majority of models converged in order to estimate the association of voice biomarkers with changes in a student’s feelings of loneliness and stress. State changes for each outcome are depicted in Multimedia Appendix 6.

Neither speech nor acoustic features were associated with acceptance or loneliness. Among students who used more positive language than their peers on average, stress was lower (OR 0.68, 95% CI 0.50‐0.92; adjusted *P*=.049) and their self-regard was higher (adjusted *P*=.04). The dichotomous self-regard analysis also showed the finding of higher word count associated with lower within-person self-regard. Table 7 contains the acceptance, loneliness, stress, and dichotomous self-regard results. Table 8 contains the continuous self-regard results.

**Table 7.** Analysis results for voice biomarkers and their association with acceptance, loneliness, stress, and self-regard^a,b^^,^^c^.

Speech features and term		Odds ratio (95% CI)		*P* value	Adjusted *P* value
Stress (0=low, 1=medium, and 2=high).					
Utterances per minute					
Student variation from mean		0.86 (0.76-0.97)		.02	.05
Student mean		1.12 (0.81-1.56)		.50	.74
VADER^d^ score					
Student variation from mean		1.14 (1.02-1.27)		.02	.05
Student mean		0.68 (0.50-0.92)		.01	.049
Self-regard (0=high self-regard and 1=low self-regard)					
Word count					
Student variation from mean		1.18 (1.04-1.34)		.009	.04
Student mean		0.82 (0.55-1.23)		.34	.45

a Analyses included 272 students (acceptance, loneliness), 113 students (stress), and 273 students (self-regard). Only features with at least one term reaching an adjusted *P*≤.05 are shown.

b Acceptance (0=low and 1=high). No features were associated with acceptance.

c Loneliness (0=high and 1=low). No features were associated with loneliness.

d VADER: Valence Aware Dictionary and Sentiment Reasoner.

**Table 8.** Analysis results for voice biomarkers and their association with continuous self-regard^a^.

Speech features and terms		Estimate (95% CI)		*P* value	Adjusted *P* value
VADER^b^ score					
Student variation from mean		0.017 (0.003 to 0.032)		.02	.12
Student mean		−0.082 (−0.136 to ‐0.028)		.003	.04

a Self-regard, continuous (lower=higher self-regard, higher=lower self-regard)

b VADER: Valence Aware Dictionary and Sentiment Reasoner.

### Daily Affect

All models evaluating the within-student and between-student associations of voice biomarkers with daily affect, measured by daily mood score, valence, arousal, and the combination of valence and arousal converged. See Table 9 for the daily affect results.

Speech features were associated with within-student negative daily affect, where a higher daily affect score was more negative. Specifically, a higher number of total utterances than usual was associated with negative daily affect (OR 1.16, 95% CI 1.09, 1.23; adjusted *P*<.001). Additionally, a negative sentiment, measured by the Valence Aware Dictionary and Sentiment Reasoner (VADER) sentiment score, was associated with negative daily affect both within-student (OR 0.92, 95% CI 0.87-0.97; adjusted *P*=.007) and between students (OR 0.87, 95% CI 0.78-0.98; adjusted *P*=.04).

Acoustic features also correlated with within-student negative daily affect. Decreased volume and physical energy than usual were associated with negative daily affect: RMS energy (OR 0.90, 95% CI 0.85-0.95; adjusted *P*<.001); magnitude mean (OR 0.83, 95% CI 0.78-0.88; adjusted *P*<.001). Furthermore, lower average pitch (OR 0.80, 95% CI 0.73-0.86; adjusted *P*<.001), pitch variance (OR 0.90, 95% CI 0.83-0.97; adjusted *P*=.01), and harmonics-to-noise ratios (OR 0.91, 95% CI 0.85-0.97; adjusted *P*=.005) were significantly associated with lower daily affect.

**Table 9.** Analysis results for voice biomarkers and their association with daily mood^a^.

Speech features and terms	Odds ratio (95% CI)	*P* value	Adjusted *P* value
Daily mood (ordinal 0=positive and 4=negative)			
Number of utterances			
Student variation from mean	1.16 (1.09-1.23)	<.001	<.001
Student mean	1.03 (0.90-1.16)	.74	.83
VADER^b^ score			
Student variation from mean	0.92 (0.87-0.97)	.003	.007
Student mean	0.87 (0.78-0.98)	.02	.04
Acoustic features			
RMS^c^ energy			
Student variation from mean	0.90 (0.85-0.95)	<.001	<.001
Student mean	0.92 (0.82-1.04)	.20	.25
Magnitude mean			
Student variation from mean	0.83 (0.78-0.88)	<.001	<.001
Student mean	0.99 (0.87-1.12)	.84	.90
HNR^d^			
Student variation from mean	0.91 (0.85-0.97)	.003	.005
Student mean	1.01 (0.87-1.16)	.94	.94
Pitch mean			
Student variation from mean	0.80 (0.73-0.86)	<.001	<.001
Student mean	1.12 (0.94-1.35)	.20	.25
Pitch variance			
Student variation from mean	0.90 (0.83-0.97)	.007	.01
Student mean	1.03 (0.86-1.24)	.75	.86
Daily mood valence (0=positive and 1=negative)			
Number of utterances			
Student variation from mean	1.16 (1.08-1.24)	<.001	.003
Student mean	1.02 (0.90-1.17)	.73	.91
Utterances per minute			
Student variation from mean	1.13 (1.05-1.21)	<.001	.01
Student mean	1.05 (0.93-1.20)	.44	.66
VADER score			
Student variation from mean	0.89 (0.83-0.95)	<.001	.007
Student mean	0.85 (0.76-0.96)	.008	.056
Acoustic features			
Student variation from mean	0.91 (0.85-0.97)	.003	.03
Student mean	0.94 (0.83-1.07)	.34	.48
Magnitude mean			
Student variation from mean	0.83 (0.77-0.89)	<.001	<.001
Student mean	1.03 (0.90-1.17)	.67	.75
HNR			
Student variation from mean	0.89 (0.82-0.96)	.002	.03
Student mean	0.99 (0.86-1.15)	.92	.98
Pitch mean			
Student variation from mean	0.78 (0.71-0.86)	<.001	<.001
Student mean	1.14 (0.95-1.38)	.17	.36
Daily mood arousal (0=low and 1=high)^e^			
Daily mood arousal and valence (0=low and negative, low and positive, high and positive, and 1=high and negative)			
Utterances per minute			
Student variation from mean	0.95 (0.87-1.04)	.29	.49
Student mean	1.23 (1.06-1.41)	.005	.046

a Analyses included 280 students. Only features with at least one term reaching an adjusted *P*≤.05 are shown.

b VADER: Valence Aware Dictionary and Sentiment Reasoner.

c RMS: root-mean-square.

d HNR: harmonics-to-noise ratio.

e No features were associated with arousal.

### Affect Valence, Arousal, and Interaction

Models fit to the 2 dimensions of core affect (valence and arousal) [16] demonstrated significant within-student associations with both speech and acoustic features, predominantly for valence but not for arousal. Consistent with the daily affect analysis, negative valence (1 vs 0 for positive valence) was significantly associated with students voicing a higher number of utterances (OR 1.16, 95% CI 1.08-1.24; adjusted *P*=.003), a higher rate of utterances per minute than usual (OR 1.13, 95% CI 1.05-1.21; adjusted *P*=.01), and by lower VADER sentiment scores than usual (OR 0.89, 95% CI 0.83-0.95; adjusted *P*=.007).

Within-student acoustic features demonstrated strong negative associations with valence. Consistent with the ordinal daily affect findings, when in a positive valence state, students voiced higher volume measured by magnitude mean (OR 0.83, 95% CI 0.77-0.89; adjusted *P*<.001), pitch, measured by pitch mean (OR 0.78, 95% CI 0.71-0.86; adjusted *P*<.001), and energy, measured by RMS energy (OR 0.91, 95% CI 0.85-0.97; adjusted *P*=.03), and harmonics-to-noise ratio (OR 0.89, 95% CI 0.82-0.96; adjusted *P*=.03) compared to their usual. No voice features were associated with arousal states.

Treating high arousal negative valence versus the 3 other states (high arousal and positive valence, low arousal and positive valence, and low arousal and negative valence), only between-student utterances per minute were associated.

## Discussion

### Principal Results

This study evaluated the utility of smartphone-captured speech and acoustic features as objective indicators of psychological states in college students. Principal findings indicate that voice biomarkers were associated with daily affect states, self-regard, and stress, but no reliable associations with loneliness or acceptance. These associations were predominantly within-student, describing how a student’s voice shifted as their own state changed over time. For self-regard and stress, only speech and linguistic features tracked these within-student shifts. Participants’ affect, derived via an ordinal label on a 5-point scale and via a binary valence label, exhibited consistent results, revealing that positive and negative core affects were characterized by a distinct speech and acoustic profile, dominated by the acoustic elements. Behaviorally, positive daily affect was associated with fewer total utterances, a lower rate of utterances per minute, and higher positive sentiment in the transcribed text. The arousal component of affect did not yield any reliable associations, while between-student utterances per min were associated with higher odds of reporting a high-activation negative state, the only reliable association observed for the valence-by-arousal interaction.

The observed discrepancy between the predictive ability of daily versus weekly models aligns with established literature on EMA and recall bias [6]. However, this difference is also driven by differences in statistical power: weekly models have fewer observations, hence fewer state changes with which to model within-student associations. When recalling contextualized emotion states such as loneliness, acceptance, stress, and self-regard over the course of a week, participants mentally aggregate and average their emotions over several days, introducing cognitive biases and inconsistencies to their responses. For instance, a high-intensity emotional experience on one day might have an outsized impact on a weekly recall in addition to temporal misalignment between the assessments. Because participants’ core affects naturally fluctuate throughout the week, the effect captured by a voice biomarker may not align with the retrospective psychological state reported each week. In contrast, daily emoji self-reports capture acute, in-the-moment affective states, and core affect in particular, which not only physically manifest in neurophysiological responses but also in the vocal biomarkers on the same calendar day of recording. This reinforces the foundational premise of EMA: capturing data in the moment reduces the cognitive biases inherent in retrospective surveys and reveals critical affective fluctuations that episodic reporting misses [6]. By pairing high-frequency voice biomarkers with high-frequency, low-burden self-reports like emojis, this study successfully uses EMA principles for modern digital phenotyping.

### Comparison With Prior Work

Our acoustic findings for negative valence are consistent with prior clinical research on vocal biomarkers. Negative daily affect was characterized by lower magnitude mean, lower pitch mean, reduced RMS energy, and lower harmonics-to-noise ratios. These features mirror lower, less energetic voice profiles classically associated with clinical depression and negative affect in previous research [12,13].

While several studies have demonstrated that the affective dimension of arousal is strongly encoded in acoustic features such as pitch and intensity [29,30], our study identified within-person associations for valence but not arousal. There are 2 explanations for this observation. First, valence and arousal were measured by a pictorial (eg, emoji-based) single momentary assessment, similar to the nonverbal self-report pictorial technique used in another study which found that arousal was more difficult to report and more error-prone [31]. Second, the evidence that arousal is strongly encoded in voice largely derives from posed, acted, or exaggerated portrayals and stimuli, which may be attenuated in a more natural setting as was studied here. Therefore, the null associations we observed for arousal may reflect both the measurement method and the natural setting rather than an absence of vocal arousal encoding.

The relationship between higher word count and low self-regard provides a unique behavioral phenotype. While previous studies [12,13] have linked clinical depression to decreased speech quantity, our findings suggest that in a college population, low self-regard may manifest behaviorally as over-explaining, or increased verbal load and processing during open-ended voice prompts, driving higher word count. This finding coheres well with research conducted on social media platforms that observes over-disclosure is tied to increased risk for mental health problems [32].

Our finding that vocal biomarkers track within-person changes in daily affect has clinical relevance based on prior literature. Daily negative affect and affect dynamics have been shown to predict depression and anxiety symptoms in young adults [33], and heightened affective instability has been shown to distinguish individuals with current depressive and anxiety disorders from those without [34]. Prior work has demonstrated that passive and active data collection using smartphones can predict clinically relevant states such as depressed mood among undergraduate college students with clinical depression [35]. Furthermore, deep learning models have been applied to longitudinal smartphone data to predict future anxiety symptoms [36]. These results position voice as a candidate signal within a broader set of biomarkers to passively assess mental health in young adults using smartphones.

### Limitations and Future Directions

Several limitations should be considered when interpreting results from this study. First off, using smartphones to collect data introduces variability and noise in derived voice biomarkers due to hardware and contextual variability. Related to voice journaling used in this study, the sample size and design did not allow us to more directly assess the impact of cultural-linguistic diversity. Second, to promote engagement, abbreviated or derived psychological measures (such as the self-regard label and single-item emoji selections) were used rather than full-length, clinically validated diagnostic interviews. This approach assesses psychological states quickly without disrupting the participants’ day, which allows for a wealth of data that is less impacted by missingness, but lacks the diagnostic depth of traditional clinical assessments. Next, this study used an observational, correlational design. While mixed-effects modeling effectively isolated the impact of within-person fluctuations and controlled for individual baselines, and care was taken to control for multiple comparisons, direct causal inferences are not possible without accompanying experimental data. Lastly, in our conservative analytical approach, several biomarkers approached but did not meet statistical significance for their association with loneliness and acceptance. Future research would benefit from more tightly aligning the time scale between voice biomarkers and psychological constructs. A larger study with a longer follow-up period would increase statistical power and allow for the application of deep-learning models. Such a study could uncover additional novel associations, including the association between affective states, psychological states (self-regard, loneliness, acceptance, and stress), and mental health diagnoses.

### Conclusions

This study demonstrates that smartphone-captured voice biomarkers can be an effective tool for monitoring changes in affective states. While voice biomarkers did not predict loneliness or acceptance evaluated at the weekly level, they were highly sensitive to weekly assessments of stress and daily fluctuations in affect and valence. The majority of associated voice features reflect how someone speaks (pitch and intensity, etc), not what they say (VADER sentiment). This study suggests that college student self-monitoring built on prosody rather than language could inform and nudge students to take the next step based on their own within-person affective changes while being less privacy-invasive than a tool that analyzes what was said. Furthermore, these voice features leverage an individual student’s changes from their own speaking rather than a comparison to other students, which furthers their perception of privacy when using such a tool. These findings underscore the importance of using high-frequency and low-friction measures such as emojis, short surveys, and voice journaling when conducting digital phenotyping, which confirms existing research [37]. Ultimately, this approach paves the way for the development of scalable, targeted interventions that act on precise mechanisms and are capable of reaching college students before symptoms escalate into clinical disorders.

## Supplementary material

10.2196/103398 Multimedia Appendix 1 List of all voice journal prompts.

10.2196/103398 Multimedia Appendix 2 AI-assisted data cleaning dialogue.

10.2196/103398 Multimedia Appendix 3 Feature correlation matrix.

10.2196/103398 Multimedia Appendix 4 *P* value table for all analyses.

10.2196/103398 Multimedia Appendix 5 Distribution of acceptance, loneliness, and stress category labels among weekly self-reports (n=2021, 2026, and 782, respectively).

10.2196/103398 Multimedia Appendix 6 Heatmaps for student state transitions for loneliness, acceptance, stress, self-regard, and daily affective states.

## Abbreviations

EMA: ecological momentary assessment
GAD-7: Generalized Anxiety Disorder 7-item scale
LLM: large language model
OR: odds ratio
PCA: principal component analysis
PHQ-8: Patient Health Questionnaire 8-item
RMS: root-mean-square
RSRB: Research Subjects Review Board
VADER: Valence Aware Dictionary and Sentiment Reasoner
