# Supplementary material for: Digital Phenotyping of Voice Biomarkers for Psychological States in College Students: Longitudinal Mobile App Study
Source: JMIR Biomed Eng. 2026 Nov 13;11:e103398. doi: 10.2196/103398 (PMC13766585; doi:10.2196/103398)

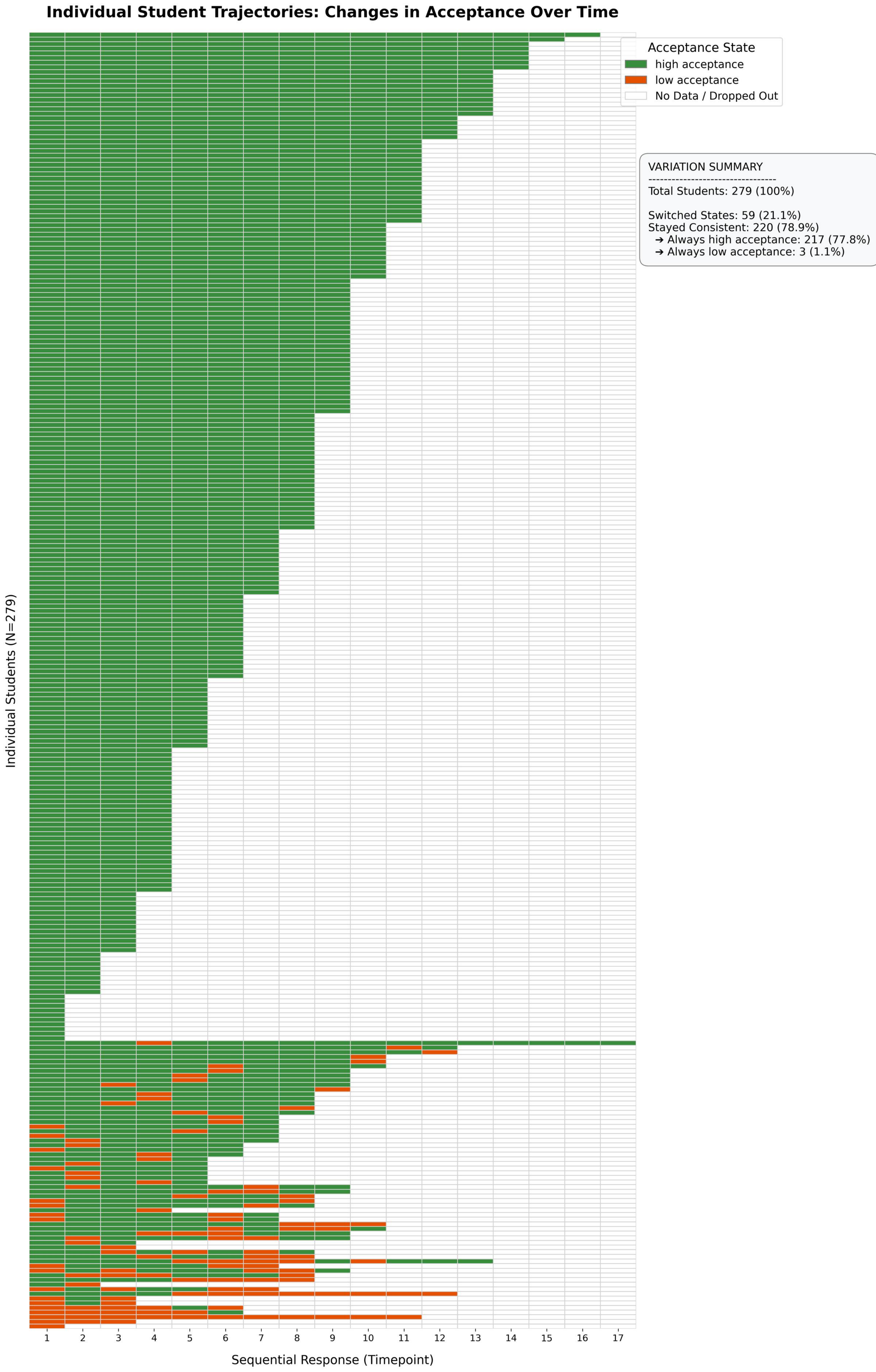

Individual Student Trajectories: Changes in Acceptance Over Time
Acceptance State
high acceptance
low acceptance
No Data / Dropped Out
VARIATION SUMMARY
Total Students: 279 (100%)
Switched States: 59 (21.1%)
Stayed Consistent: 220 (78.9%)
➔ Always high acceptance: 217 (77.8%)
➔ Always low acceptance: 3 (1.1%)
Individual Students (N=279)
1
2
3
4
5
6
7
8
9
10
11
12
13
14
15
16
17
Sequential Response (Timepoint)

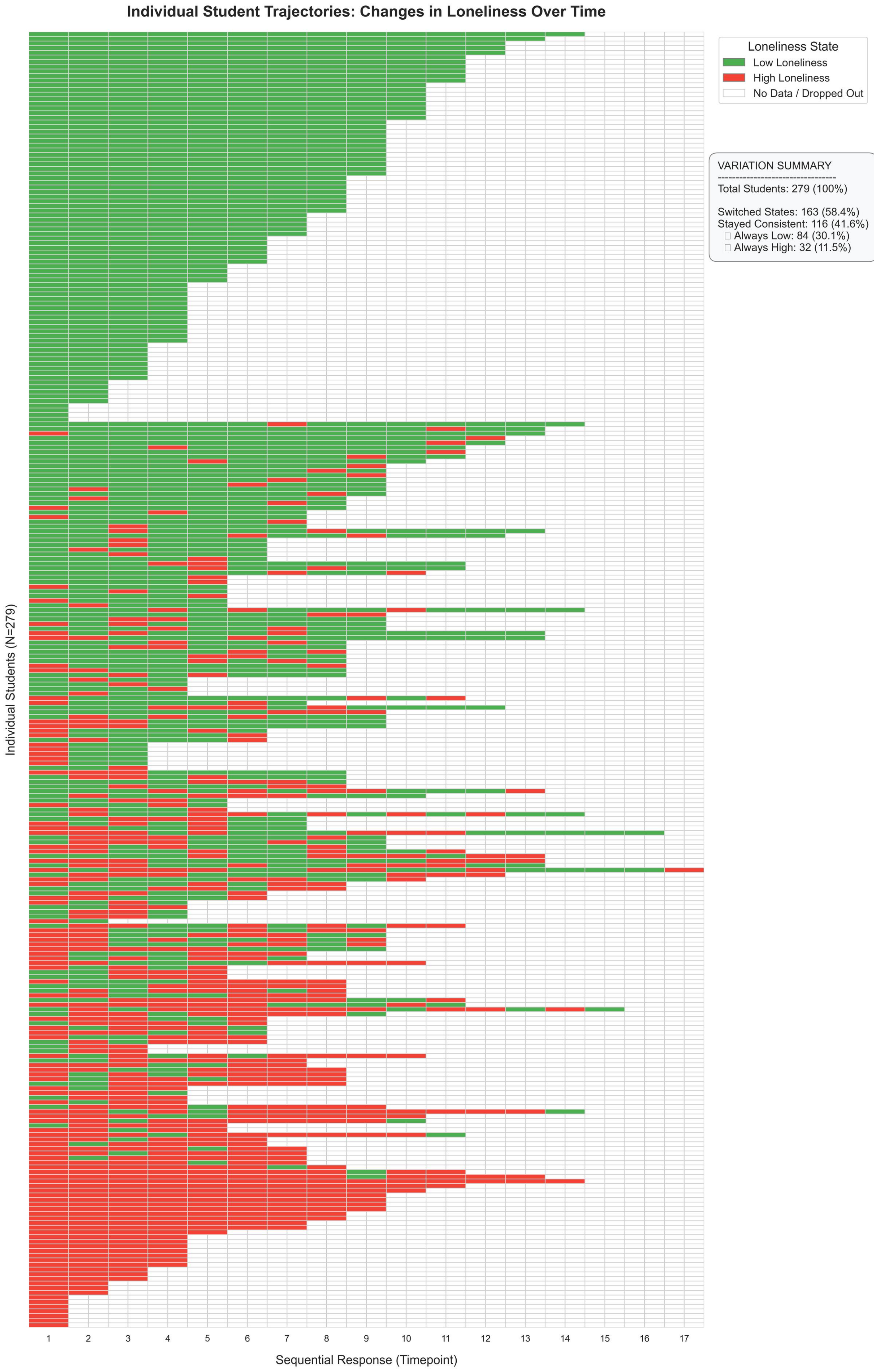
Individual Student Trajectories: Changes in Loneliness Over Time
Loneliness State
Low Loneliness
High Loneliness
No Data / Dropped Out
VARIATION SUMMARY

Total Students: 279 (100%)
Switched States: 163 (58.4%)
Stayed Consistent: 116 (41.6%)
Always Low: 84 (30.1%)
Always High: 32 (11.5%)
Individual Students (N=279)
1
2
3
4
5
6
7
8
9
10
11
12
13
14
15
16
17
Sequential Response (Timepoint)

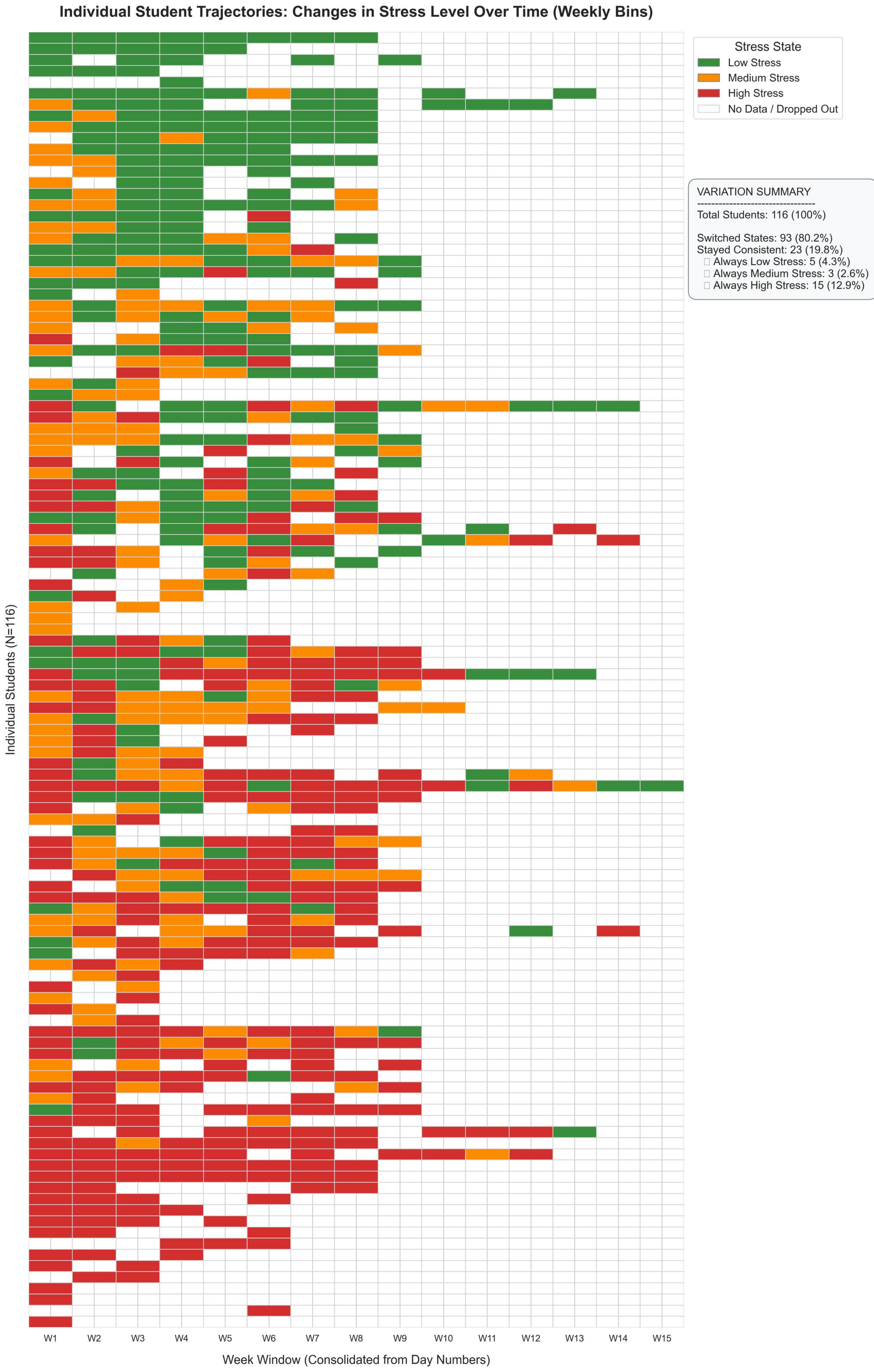
Individual Student Trajectories: Changes in Stress Level Over Time (Weekly Bins)
Stress State
Low Stress
Medium Stress
High Stress
No Data / Dropped Out
VARIATION SUMMARY

Total Students: 116 (100%)
Switched States: 93 (80.2%)
Stayed Consistent: 23 (19.8%)
Always Low Stress: 5 (4.3%)
Always Medium Stress: 3 (2.6%)
Always High Stress: 15 (12.9%)
Individual Students (N=116)
W1
W2
W3
W4
W5
W6
W7
W8
W9
W10
W11
W12
W13
W14
W15
Week Window (Consolidated from Day Numbers)

Individual Student Trajectories: Changes in Self-Regard Over Time

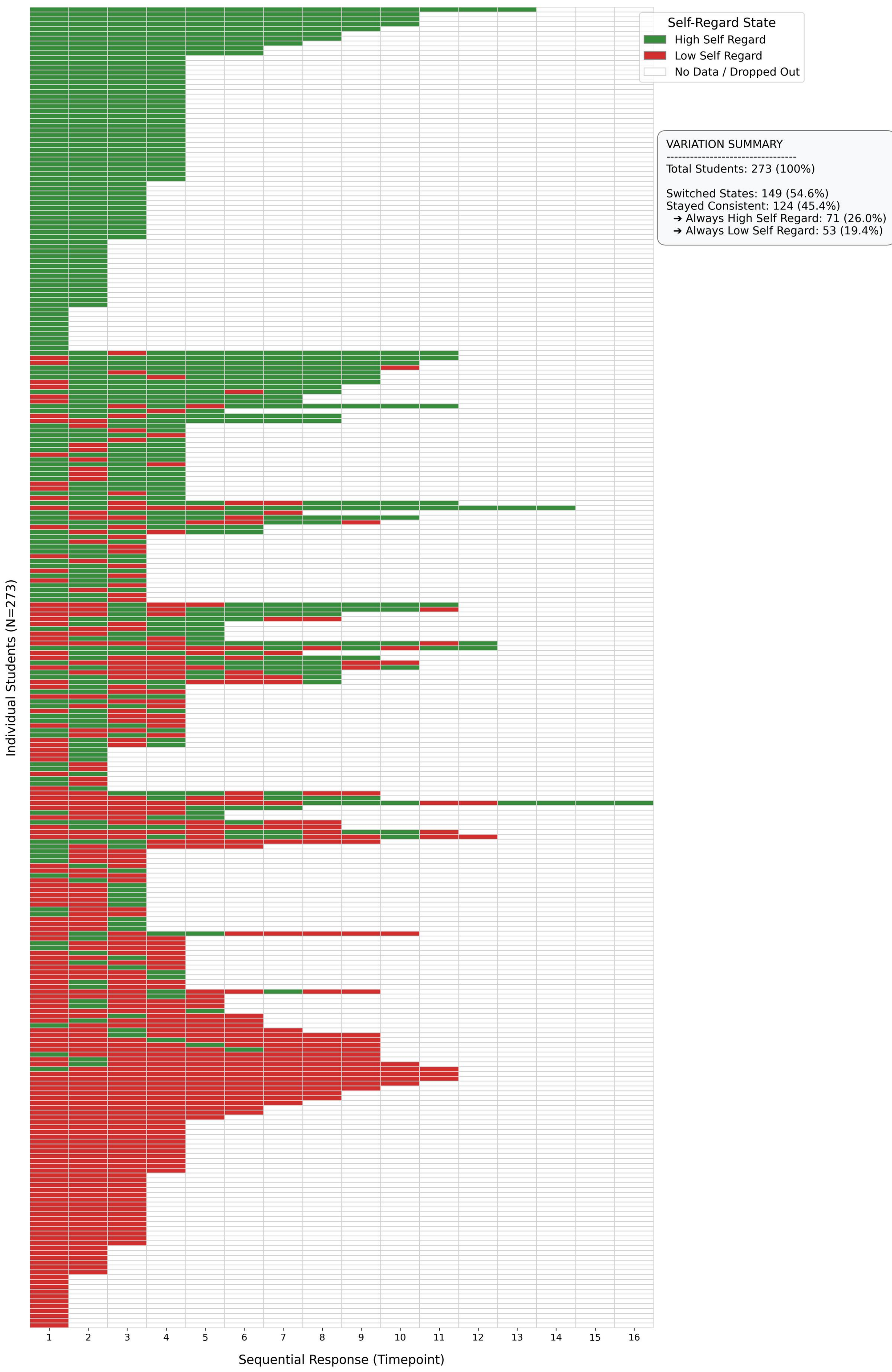

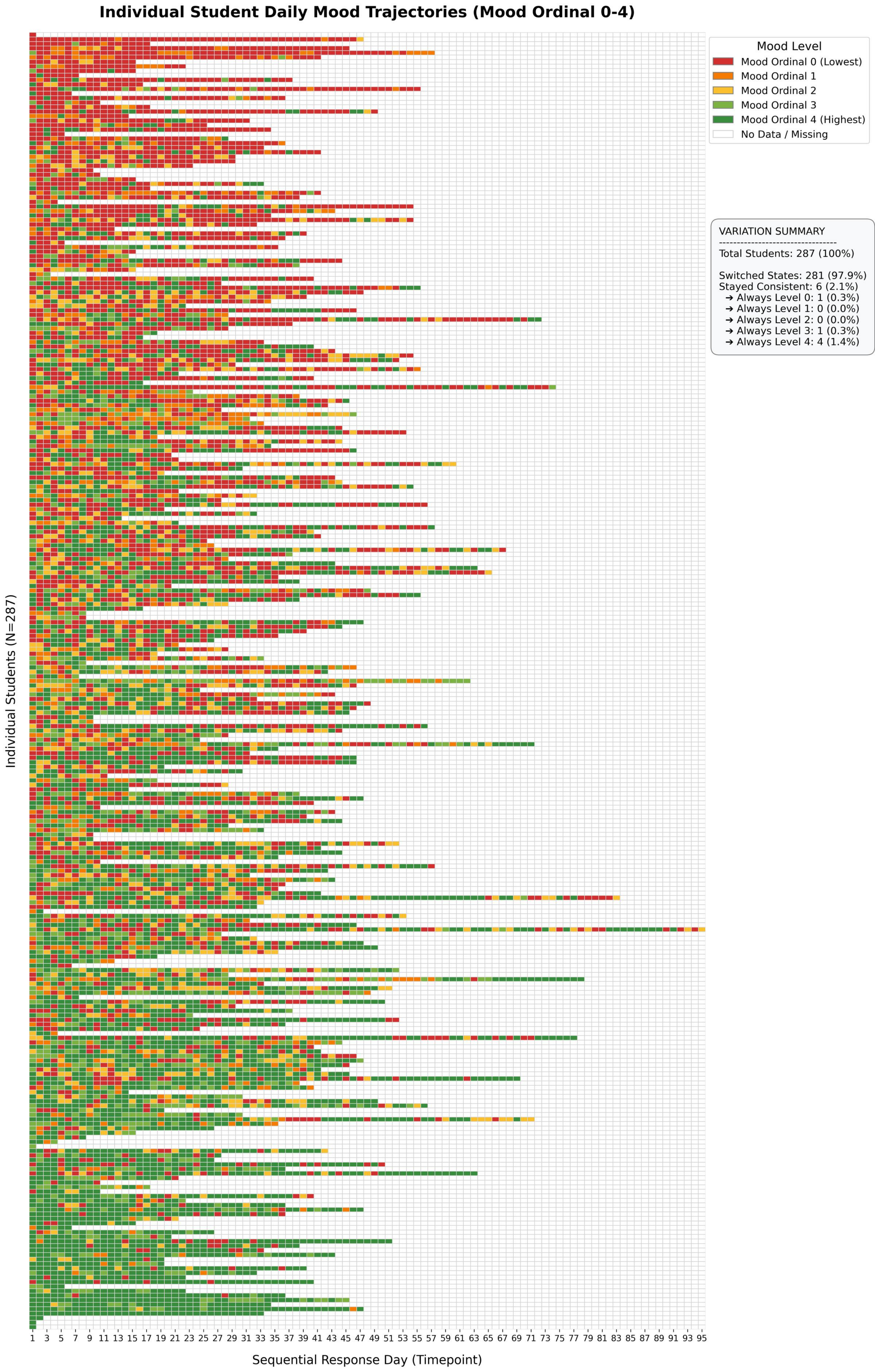

Individual Student Daily Mood Trajectories (Mood Ordinal 0-4)
Mood Level
Mood Ordinal 0 (Lowest)
Mood Ordinal 1
Mood Ordinal 2
Mood Ordinal 3
Mood Ordinal 4 (Highest)
No Data / Missing
VARIATION SUMMARY

Total Students: 287 (100%)
Switched States: 281 (97.9%)
Stayed Consistent: 6 (2.1%)
➔ Always Level 0: 1 (0.3%)
➔ Always Level 1: 0 (0.0%)
➔ Always Level 2: 0 (0.0%)
➔ Always Level 3: 1 (0.3%)
➔ Always Level 4: 4 (1.4%)
Individual Students (N=287)
1 3 5 7 9 11 13 15 17 19 21 23 25 27 29 31 33 35 37 39 41 43 45 47 49 51 53 55 57 59 61 63 65 67 69 71 73 75 77 79 81 83 85 87 89 91 93 95
Sequential Response Day (Timepoint)

Individual Student Daily Trajectories: Valence_1 Over Time

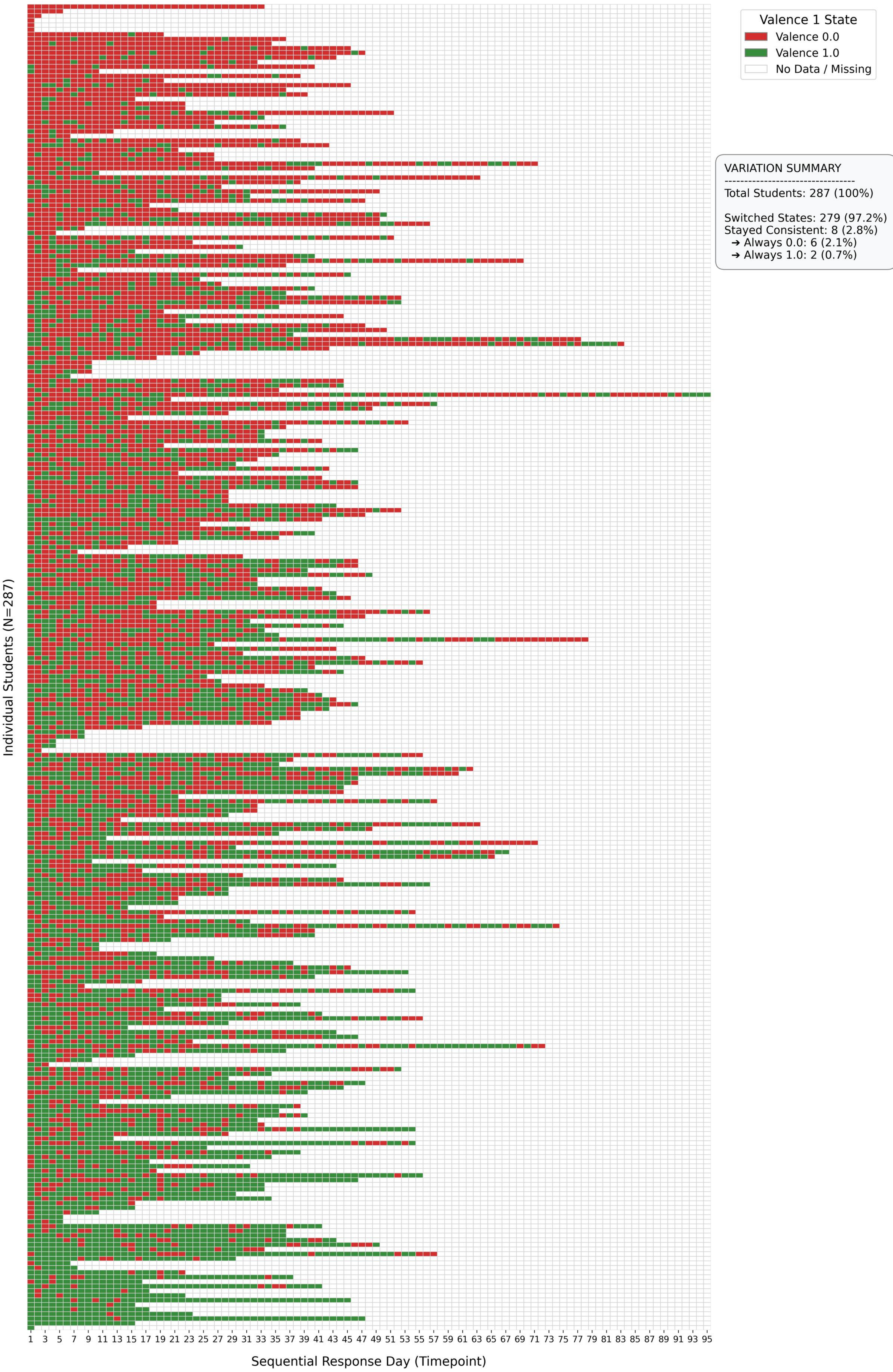

Individual Student Daily Trajectories: Arousal_1 Over Time

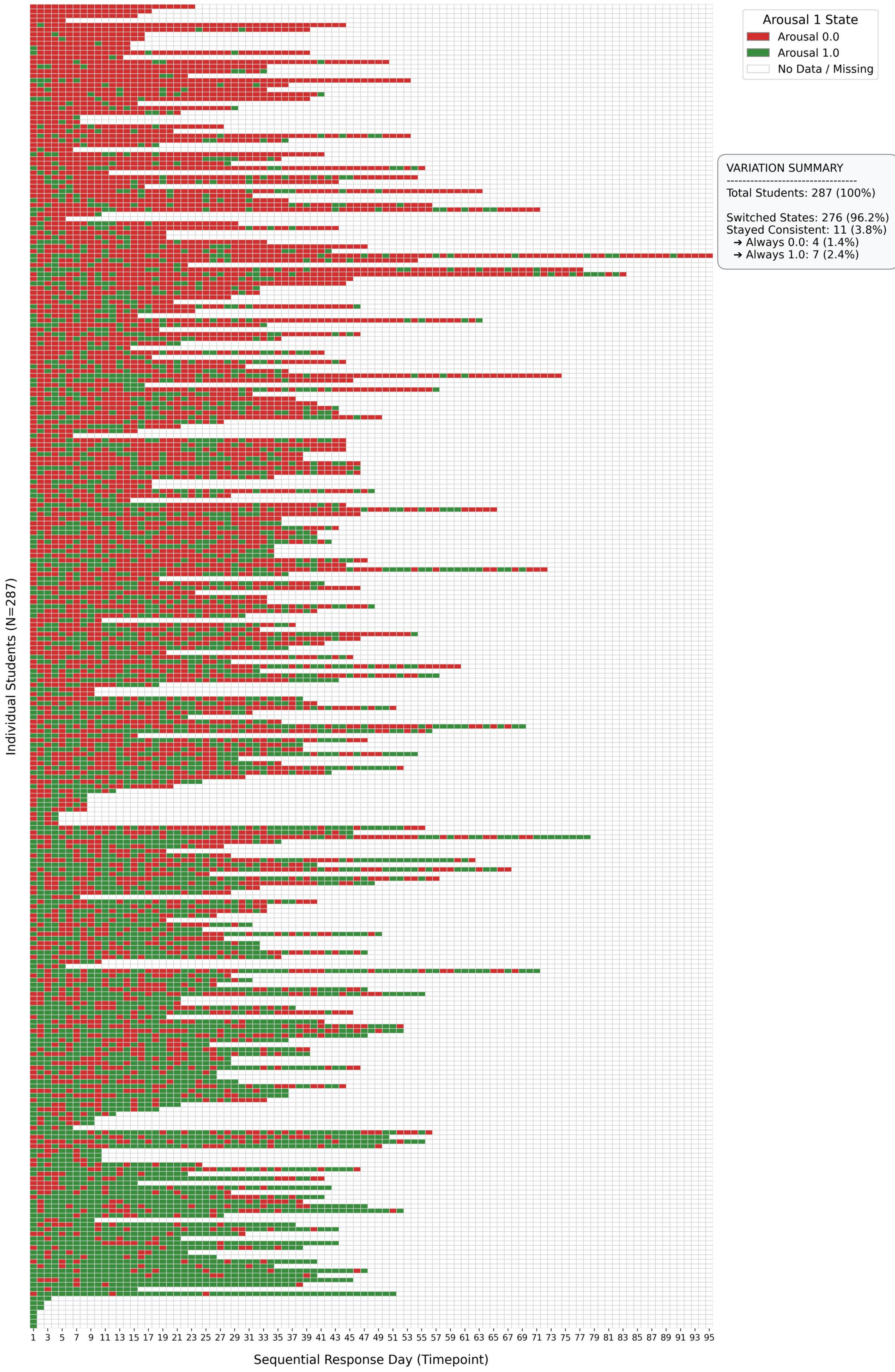

Supplement: Supplementary file 2 [file biomedeng-v11-e103398-s006.pdf]
